# Comparison of Bacterial Populations in the Ceca of Swine at Two Different Stages and Their Functional Annotations

**DOI:** 10.3390/genes10050382

**Published:** 2019-05-20

**Authors:** Himansu Kumar, Woncheol Park, Krishnamoorthy Srikanth, Bong-Hwan Choi, Eun-Seok Cho, Kyung-Tai Lee, Jun-Mo Kim, Kwangmin Kim, Junhyung Park, Dajeong Lim, Jong-Eun Park

**Affiliations:** 1 Division of Animal Genomics and Bioinformatics, National Institute of Animal Science, RDA, Wanju 55365, Korea; himanshu.genetics@gmail.com (H.K.); wcpark1982@korea.kr (W.P.); kris87@korea.kr (K.S.); bhchoi@korea.kr (B.-H.C.); djlim@korea.kr (D.L.); 2 Swine Science Division, National Institute of Animal Science, RDA, Cheonan 31000, Korea; segi0486@korea.kr; 3 Animal Genetics and Breeding Division, National Institute of Animal Science, RDA, Cheonan 31000, Korea; leekt@korea.kr; 4 Department of Animal Science and Technology, Chung-Ang University, Anseong 17546, Korea; junmokim@cau.ac.kr; 5 3 BIGS CO. LTD., Hwaseong 18454, Korea; kmkim@3bigs.com (K.K.); jhpark@3bigs.com (J.P.)

**Keywords:** cecum microbiota, 16S rRNA sequencing, SILVA database

## Abstract

The microbial composition in the cecum of pig influences host health, immunity, nutrient digestion, and feeding requirements significantly. Advancements in metagenome sequencing technologies such as 16S rRNAs have made it possible to explore cecum microbial population. In this study, we performed a comparative analysis of cecum microbiota of crossbred Korean native pigs at two different growth stages (stage L = 10 weeks, and stage LD = 26 weeks) using 16S rRNA sequencing technology. Our results revealed remarkable differences in microbial composition, α and β diversity, and differential abundance between the two stages. Phylum composition analysis with respect to SILVA132 database showed *Firmicutes* to be present at 51.87% and 48.76% in stages L and LD, respectively. Similarly, *Bacteroidetes* were present at 37.28% and 45.98% in L and LD, respectively. The genera *Prevotella*, *Anaerovibrio*, *Succinivibrio*, *Megasphaera* were differentially enriched in stage L, whereas *Clostridium*, *Terrisporobacter*, *Rikenellaceae* were enriched in stage LD. Functional annotation of microbiome by level-three KEGG (Kyoto Encyclopedia of Genes and Genomes) pathway analysis revealed that glycine, serine, threonine, valine, leucine, isoleucine arginine, proline, and tryptophan metabolism were differentially enriched in stage L, whereas alanine, aspartate, glutamate, cysteine, methionine, phenylalanine, tyrosine, and tryptophan biosynthesis metabolism were differentially enriched in stage LD. Through machine-learning approaches such as LEfSe (linear discriminant analysis effect size), random forest, and Pearson’s correlation, we found pathways such as amino acid metabolism, transport systems, and genetic regulation of metabolism are commonly enriched in both stages. Our findings suggest that the bacterial compositions in cecum content of pigs are heavily involved in their nutrient digestion process. This study may help to meet the demand of human food and can play significant roles in medicinal application.

## 1. Introduction

Pigs are principally farmed to meet the demand of human food but also useful in clothing, cosmetics, processed food ingredients, and medicinal purposes [1]. Numerous studies have shown that the microbial community plays a significant role in multiple functions of the host, for example food digestion, absorption, and the immune system [2]. It was reported that around 1014 bacterial populations encompassing 500–1000 bacterial species are present in the mammalian gastrointestinal tract (GIT) [3,4]. Previously, the gut microbiota of mammals or pigs was studied by culture-dependent method [5]. However, due to unknown growth requirements of various species of bacteria, the method was not supposed to be sufficient to explore the microbial population [1]. Multiple advanced techniques such as denaturing gradient gel electrophoresis (DGGE) [6,7], terminal-restriction fragment length polymorphism (T-RFLP) [8], temporal temperature gradient gel electrophoresis (TTGE) [9], and Sanger sequencing [10] have been introduced to decipher the gut microbiota. Although those techniques are more robust than microbial culture-dependent ones, they still lack the coverage of the diverse microbial community [11]. Recently, a 16s rRNA high-throughput next-generation sequencing technique with high coverage diversity was introduced for microbial population study [12]. Since then, 16s rRNA genes are well known and used to classify and identify microbes, because of their ubiquitous presence in microbes. Apart from the NCBI (National Center for Biotechnology Information) database, secondary databases specifically for 16s rRNA genes, such as SILVA and Greengenes are being developed [13,14]. SILVA consists of quality-checked, updated, and comprehensive datasets of aligned small 16S rRNA sequences for *Bacteria*, *Archaea*, and *Eukarya* [13], whereas Greengenes is a taxonomy database based on de novo phylogeny that provides comprehensive 16s reference datasets [14].

The GIT microbiome of pig plays essential roles, such as a pathogen barrier, and the microbial population in cecum reveals the correlation between microbiomes and metabolites [15,16]. It has been proven that the microbial communities of GIT are changing from childhood to adulthood, and dominated by bacteria. Kim et al. in 2011 reported that the microbiome population changes from neonates to adults as aerobes to anaerobes, respectively [17]. Multiple machine-learning algorithms have been proposed for taxonomical classifications as well as functional annotation [18,19,20,21]. Most recently, Fiannaca et al., [22] has introduced deep-learning models for microbial taxonomic classification from 16 s shotgun and amplicon metagenomic sequencing data. Furthermore, Maltecca, Christian et al., has also predicted the growth and carcass traits of swine through Bayesian, random forest, gradient boosting etc. [23].

In this study, we have compared the bacterial composition in the ceca of pigs at different ages (10 weeks, L; 26 weeks, LD) through 16s rRNAs sequencing [24,25]. Pigs from weaning to 10 weeks are termed as weaners and they experience numerous changes in their life such as changes in diet, stress of being separated from the sow, environmental changes, etc. It has been noticed that microbial populations of pigs of <10 weeks of age are dynamically changing and, in contrast to pigs between 10 and 26 weeks, the cecum microbiota is relatively stable. We have used 16 animals to collect the sample, 8 from each stage. Each sequence assigned an operational taxonomy unit (OTU) based on alignment with the databases SILVA. Furthermore, functional annotation of microbial sequences was also done with the help of PICRUST (Phylogenetic Investigation of Communities by Reconstruction of Unobserved States) [26] and through machine-learning approaches such as LEfSe, random forest, and Pearson’s correlation. Multivariate analysis was also done through principal component analysis (PCA) [27], redundancy analysis (RDA) [28], canonical correspondence analysis (CCA) [29], and detrended correspondence analysis (DCA) [30] to explore the ecological association. We performed a Pearson’s correlation network analysis to establish the relationship among identified functions. Comprehensive study of microbial interactions at different time points can help to implement the strategy to improve age-related pig farming as well as the well-being of swine [31,32].

## 2. Materials and Methods

### 2.1. Ethics Statement, Animal Rearing, and Feeding

A total of 16 F1 crossbred pigs between Korean native and Yorkshire breed were used in this study. The experiment was reviewed and approved by the Institutional Animal Care and Use Committee, National Institute of Animal Science, South Korea (IACUC no. NIAS2016-848). Feed and water were supplied ad-libitum during the experiment. These piglets were randomly allocated into two groups, raised for 10 weeks and 26 weeks. Out of 16 piglets, 8 of them were male and 8 were female. Feeding components as commercial formula diet for each stage is shown in Table 1.

### 2.2. Sample Collection, Nucleic Acid Extraction, and 16s rRNA High-Throughput Sequencing

The animals were sacrificed at the end of the growth stage and samples were collected from cecum. The experimental area was cleaned and disinfected before every sample collection to avoid cross-contamination among samples. All collected samples were transferred to liquid nitrogen temporarily and sent to lab for storage at −80 °C. The total microbial DNA of all the samples were following the standard protocol of the DNA isolation kit (Qiagen, Germany) and quality of the extracts were checked using NanoDrop (Thermo scientific, Waltham, MA, USA). Good-quality samples (according to the ratio of absorbance at 260/280 nm) were used for library preparation. During all experimental procedure, pigs were not treated with any antibiotics. V4–V5 hyper-variable regions have been amplified by using universal primers of bacterial 16S rRNA gene. For the use of PCR, samples were diluted as 1:10, subsequently, the universal primer was used as 515F (5′-GTGCCAGCMGCCGCGGTAA) and 806R (5′-GGACTACHVGGGTWTCTAAT). PCR products were purified and used to construct the library and sequenced on MiSeq platform (Illumina, San Diego, CA, USA) [33].

### 2.3. Sequence Quality, Assembly, Preprocessing, and Clustering

FLASH 1.2.11 (Fast Length Adjustment of Short Reads) assembly program was used for assembly [34]. It is a fast and accurate tool to merge paired end reads from next-generation sequencing experiments [35,36]. Short reads were filtered out and extra-long tails were trimmed. Filtered reads were clustered at 100% identity using CD-HIT-DUP [37] which provided an OTU. It uses three-step clustering methods, first as raw read filtering and trimming [38], second error-free reads picking, and at the last step, it clusters at different distance cutoffs (0.03). The rDnaTools is a Python package from the MOTHUR suit of utilities for working with rDNA sequences data generated by PacBio SMRT sequencing technology [39]. Currently, rDnaTools implements a single pipeline for the export, filtering, and cluster of 16S sequences [40].

By using CD-HIT-DUP and RDnaTools, chimeric reads were identified [41]. Secondary clusters are recruited into primary clusters. Noise sequences such as ambiguous base detection, miss-matched primer, extra-long read, etc. were removed from the cluster, and the remaining representative reads were clustered using a greedy algorithm and assigned OTU at a user-specific OTU cutoff (e.g., 97% ID at species level) [42].

### 2.4. Taxonomic Assignment and Diversity Analysis

QIIME (v1.91) is known bioinformatics pipeline to analyze the microbial samples, was used to assemble the paired end reads into tags according to their sequence overlap relationship [43]. Preprocessing consisted of removal of primers, demultiplexing and quality filtering (Phred ≥ 20), chimera removal, etc. by using usearch61 module [44]. We used Silva132 database (April 2018 release) to pick up the OTU using the open-reference analysis method, allocated the taxonomy by using the uclust, created the phylogenetic tree by using QIIME FastTree, and checked the OTU identifier [13,41]. After filtering, an OTU table was generated in biom format [45]. The resulting sequences were clustered into OTUs based on a similarity threshold of ≥97% by PyNAST [46]. We performed comparative OTU assignment against the database at phylum, class, order, family, genus, and species levels. We used *α diversity.py* script in QIIME to analyze the α-diversity to understand the local population of the microbiome. Multiple algorithms such as ACE (abundance-based coverage estimator), Chao1 (richness estimator), Observed_otus (distinct OTUs estimator), Shannon (entropy estimator), and Simpson (Simpson’s index calculator) were used to estimate the α diversity. The rarefaction curves of α diversity were visualized with the help of Phyloseq v1.8.2 of R Package [47].

*β diversity.py* of QIIME was used to calculate β diversity for estimating the correlation among other factors and microbes. Microbial distribution was analyzed by the principal coordinate analysis (PCoA) and 2D plots were produced with the help of *make_2d_plots.py* [48]. Phylogenies among identified OTUs were calculated by unweighted pair-group method using arithmetic averages (UPGMA) clustering techniques. To calculate the robustness of individual UPGMA clusters and PCoA plot cluster, jackknife β diversity program was also used [49].

### 2.5. Bioinformatics Analysis for Functional Annotations

Multiple bioinformatics approaches such as multivariate statistical analysis, machine learning, PICRUST, STAMP, and Pearson’s correlation were used to annotate the functions of bacterial compositions present in the cecum of pigs. Differential abundance of microbial functions has also been analyzed.

#### 2.5.1. Multivariate Statistical Analysis to Explore Complex Ecological Associations

In this study, we have used multivariate statistical tools such as PCA [27], redundancy analysis (RDA) [28], canonical correspondence analysis (CCA) [29], detrended correspondence analysis (DCA) [30], and non-metric multidimensional scaling (NMDS) [50] to explore the microbial community composition and other explanatory variables. Furthermore, the mixMC (mixOmics microbial community) has also been used. It provides access to multivariate methods and is implemented in mixMC R package. It is a multivariate framework that takes into account the sparsity and compositionality of microbiome data. mixMC aims to identify specific associations between microbial communities and explanatory variables, such as habitat [51]. It builds on the hypothesis that multivariate methods can help identify microbial communities that modulate and influence biological systems as a whole.

#### 2.5.2. Comparative Functional Annotation of Cecum Content

Functional studies have been done on the basis of phylogenetic investigations of communities by reconstructing the unobserved states using PICRUST [26]. Relative abundance of phyla, class, order, genus, and species were estimated with the help of PICRUST and STAMP program [52,53]. Multivariate analysis between two growth stages (L vs. LD) was done with the help of PCA, whereas univariate analysis was done with the help of an ANOVA (analysis of variance) test [54]. Random forest [55], a robust machine-learning algorithm was used to describe the metagenome and their involvement in metabolic processes.

#### 2.5.3. Network Analysis through Pearson’s Correlation

Network analysis has been done to identify the co-occurring and mutually exclusive microbial functions through pathway analysis. Pathways are represented as nodes, and its abundance as node size, and edges represent positive and negative associations [56]. Nodes can be colored based on their association with selected environmental variables (Pearson’s correlation) [57].

## 3. Results

### 3.1. Sequence Analysis of Cecum Content

All 16 pigs (L = 8, LD = 8) were raised separately and their body weight and feed intake were recorded and regulated daily. All sequences were generated by 16S rRNA sequencing technology of F1. Crossbreed Korean native x Yorkshire pigs are shown in Table 2, as total bases, read count, GC percentage, Q20, Q30 values, etc.

FATSQC-approved sequencing reads of each sample are assembled and shown in Table 2. The DNA sequences of all samples were pooled to calculate the microbial diversity by Shanon–Weaver and Simpson indices. As shown in Table 3, γ-diversity represents the diversity across an entire landscape (α + β diversity). α-diversity corresponds to species diversity in sites/habitats at a local scale and β-diversity comprises species diversity among sites or habitats as shown in Table 3. We used 10 iterations for calculation of α diversity and kept a 39,304 sequence depth for calculation of metrices. The α diversity metrics (*p*-value) Excel file is provided in the Supplementary Material (Supplimentary_Excel_Sheet_1). We kept 97% sequence similarity to get an OTU, an operational definition of a species or group of species often used when only DNA sequence data is available. Chao1 returns the richness estimate for an OTU. The Shannon index takes into account the number and evenness of species, and the Simpson index represents the probability that two randomly selected individuals in the habitat will belong to the same species. Goods coverage is calculated as C = 1-(s/n), where s is the number of unique OTUs and n is the number of individuals in the sample. This index gives a relative measure of how well the sample represents the larger environment. As shown in Supplementary Figure S1a–c, few differences in chao1, Shannon, and Simpson were observed between the two stages (L vs. LD). However, the difference between the two groups—core microbiome and the group taxonomy results—can be seen. In particular, in the case of coremicrobiomes, the core OTU is 519 and the unique OTU is 102 among 621. *Ruminobacter* (0.40%), *Mitsuokella* (0.38%), *Lactobacillus*_CP000033.434247.435818 (0.12%), and *Veillonella* (0.08%) are at the genus level, whereas, principle coordinate plot, based on weighed unifrac distance matrices and β diversity analysis, was significantly clustered as L and LD, shown in Supplementary Figure S1d.

### 3.2. Taxonomic Assignment

**Figure 1:**
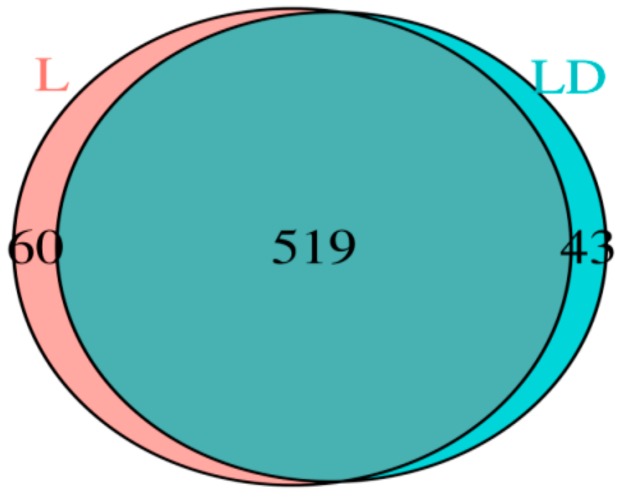
Venn diagram of unique and shared OTUs between L and LD stages.

**Figure 2:**
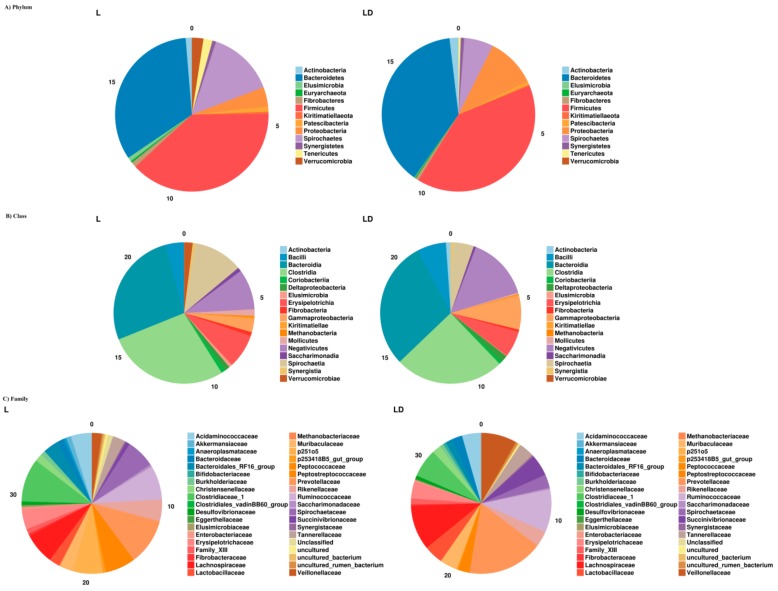
Pie chart presentation of taxonomic proportions (**A**) phylum- (**B**) class- (**C**) family-level classification.

We conducted stage wise taxonomy analysis of microbial composition by using RDP (Ribosomal Database Project) classifier, phylum, class, and family-level taxonomic annotations. Out of a total of 47 family, 158 genus, and 287 species sequences, unclassified reads were 7, 30, and 239, respectively; a complete list of taxonomic abundance percentages are provided in the Supplementary Material (Supplimentary_Excel_Sheet_2). Overall, a major portion (>90%) of the bacterial composition belongs to *Bacteroidetes* and *Firmicutes*. Interestingly, the composition of *Bacteroidetes* increases from 37.28% to 45.98% with the age of pigs (L vs. LD), whereas the composition of *Firmicutes* decreases from 51.87% to 48.76% as shown in Figure 1. Furthermore, we investigated the changes among other microbial taxonomy distributions, such as the composition of Spirochaetes, which decreases from 9.13% to 1.19%. However, composition of Protobacteria increases from 0.71% to 3.78%. Genus-level comparison shows that *Anaerovibrio* is present as the highest population at LD stage as 7.53%, as compared to L as 0.94%. *AlloPrevotella* is present as 2.79% in L and 0.92% in LD. A Venn diagram of unique and shared OTUs between L and LD stages has been shown in Figure 1. A total of 519 OTUs were common in both growth stages. However, 60 and 43 OTUs are unique in L and LD, respectively. A complete Excel sheet containing the phylum-, class-, order-, family-, genus-, and species-wise population has been provided in the Supplementary Data (Supplimentary_Excel_Sheet_3). The overall composition of microbiota has been shown in Figure 2.

### 3.3. Genus-Level Univariate Analysis

**Figure 3:**
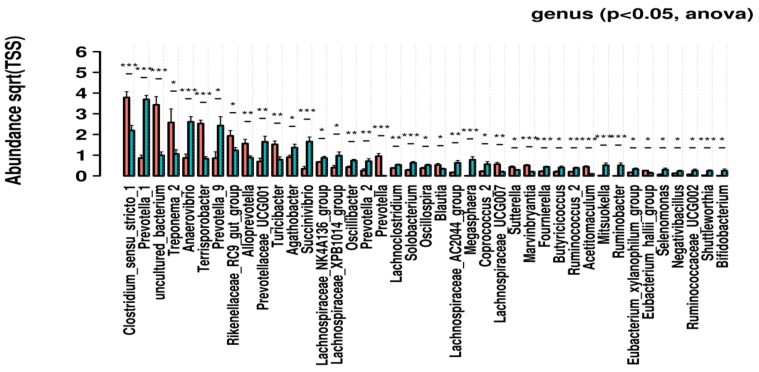
Genus-level univariate analysis through ANOVA test, Red color indicates the stage L, and blue is stage LD. *X*-axis representing the samples and *Y*-axis representing the relative OTU counts. * *p* ≤ 0.05, ** *p* ≤ 0.01, *** *p* ≤ 0.001, **** *p* ≤ 0.0001.

We conducted univariate analysis by ANOVA for identifying genus-level abundance. The significantly different genus is shown in a bar chart, and standard error is represented by error bars. The t-test was done for pairwise comparison as shown in Figure 3. The *p*-value threshold for significance was than 0.05 and found that some of the genera such as *Clostridium*, uncultured bacterium, *rikenellaceae*, *Prevotellaceae*, etc. are highly abundant in stage L, whereas in stage LD *Prevotella_1*, *Succinivibrio*, *Roseburla*, *Anaerovirio*, etc. are differentially abundant. The ANOVA plots for phylum, class, family, order, and species levels are shown in Supplimentary Figure S2a–d.

### 3.4. Statistical Analysis for Functional Annotation

**Figure 4:**
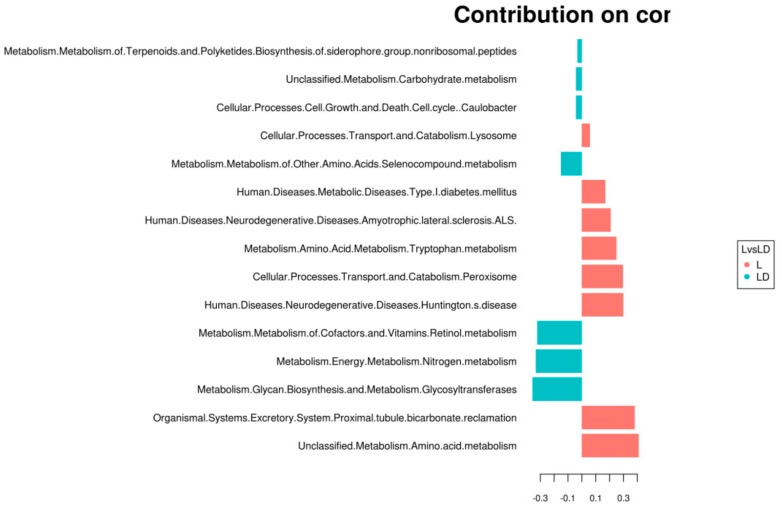
Sparse partial least discriminant analysis, association contribution plot showing pathway association between two stages.

We have used mixMC for this study, as mixMC is being used extensively for microbial biomarker discovery [51]. It is a multivariate framework which can take into account sparsity and composition of the metagenomic data and establish associations between microbial populations and their habitat. The mixMC uses principal component analysis (PCA), and sparse partial least discriminant analysis (sPLS-DA). To predict the most prominent functional pathway that can describe best functional behavior of microbes present in the two different age groups of swine, we used sparse partial least discriminant analysis. We conducted analysis of the top 100 most abundant pathways present at ≥2 time points. As shown in Figure 4 (blue color indicates stage LD, and red color indicates stage L), the contribution plot shows the OTUs associated with both stages. The most consistently abundant pathways were compared with the metagenome sequence and shown as a violin plot. Amino acid metabolic pathways (glycine, serine, threonine, arginie, and proline) are highly abundant in stage L, whereas glycan biosynthesis, carbon fixation, sulfur metabolism, nitrogen metabolism, and genetic information are highly abundant in stage LD. Other associated plots such as PCA, CCA, NMDS, and RDA are shown in Supplementary Figure S3a–d. Functional profiling of a microbial community has been inferred on the basis of marker genes present in one or more samples. The OTU table represented the gene sequences of the marker gene with its relative abundance in each of the samples. Some of the stage-specific abundant metabolic pathways are shown in Figure 4.

**Figure 5:**
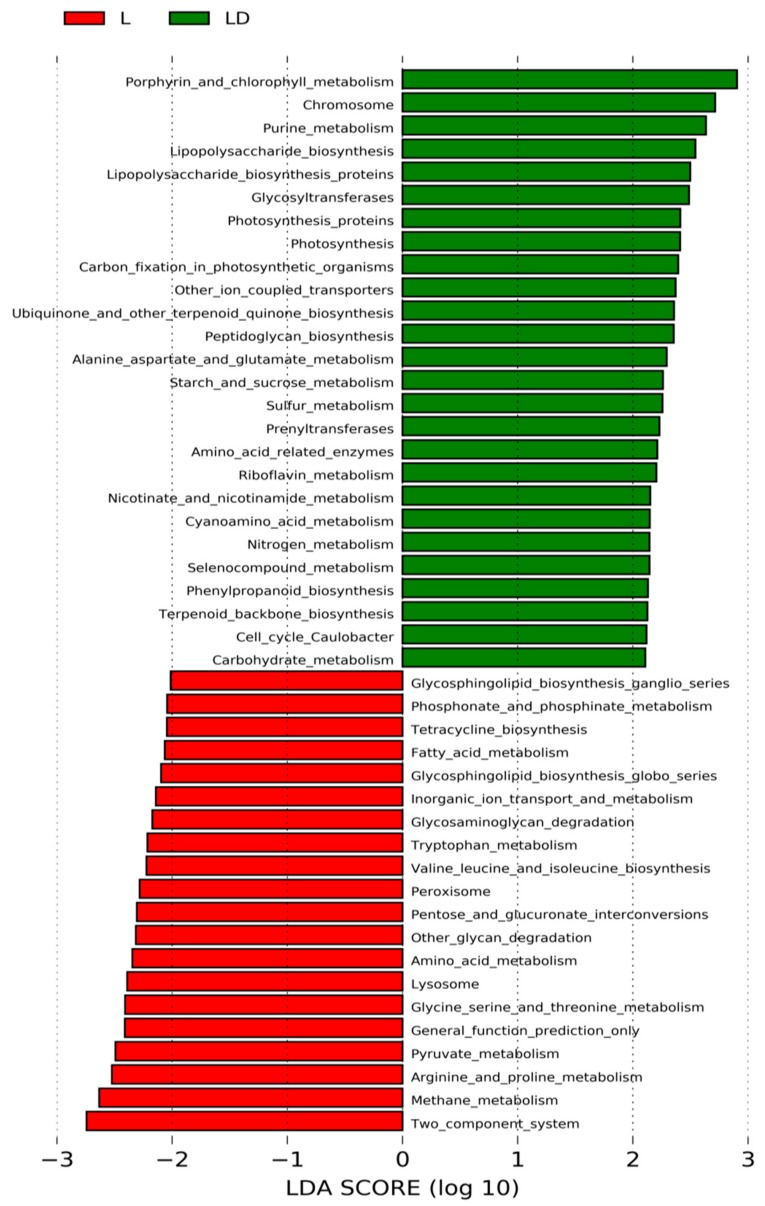
Comparative functional analysis through linear discriminant analysis (LDA) effect size (LEfSe) of microbiota of both stages. Histogram representation of LDA scores ((log10) > 2) have been computed for differentially abundant pathways between the L and LD stage.

We have used linear discriminant analysis (LDA) effect size (LEfSe) algorithm and standard statistical tests to determine the functions of the OTUs. Based on LDA score, functional abundance has been inferred for both stages (Supplimentary_Excel_Sheet_4). LDA is similar to ANOVA and regression analysis, which attempts to produce one dependent variable as a linear combination. Differential abundances of selected metabolic activity and their associations with stages L and LD through ANOVA analysis is shown in Supplementary Figure S4. Comparative analysis of functional profiles through LEfSe shown in Figure 5, in stage LD, porphyrine and chlorophyll metabolism, lipopolysaccharide biosynthesis, photosynthesis etc. are highly enriched, while in stage L, lysosome, two-component systems, and amino acid metabolism are highly enriched. Furthermore, random forest (RF) method was used for functional profiling through feature selection. RF is an ensemble classification algorithm, which can combine the regression, by averaging the results of all trees and predicted the class as a reflection of all class output. RF predicts that the pathways such as secretion system, DNA replication, two-component systems, bifolate metabolism, fructose and mannose metabolism, ABC (ATP-binding cassette) transporter systems, etc., are highly enriched (Supplementary Figure S5).

**Figure 6:**
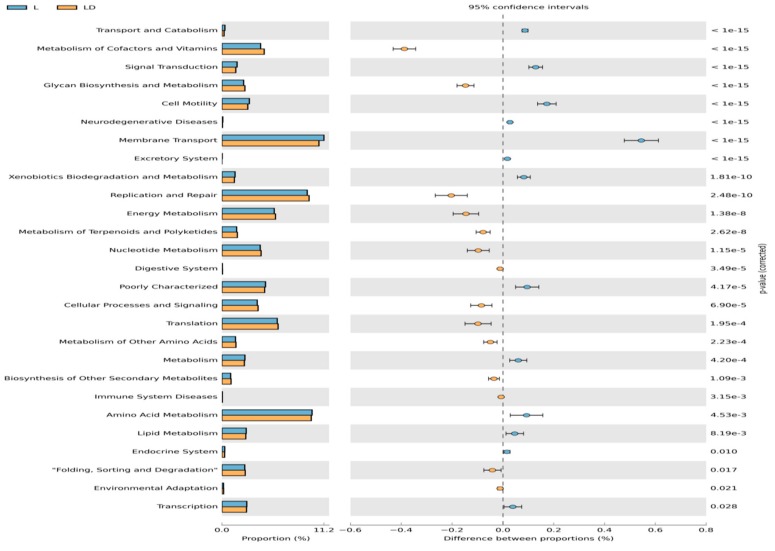
Mean proportion and their differences in predicted functional metagenomes of the cecum microbiota. Blue color indicates stage L and yellow indicates stage LD.

Comparative functional metagenome profiling through KEGG ontology between two stages of cecum content has also been established through STAMP software. As shown in Figure 6, purine metabolism is differentially enriched in stage L, whereas ABC-2-type transport systems, HPr kinase/phosphorylase, and glycolytic pathways are differentially enriched in stage LD at 95% confidence intervals. Some other metabolic pathways such as HPr kinase, glucokinase, β-glucosidase, etc. were differentially enriched in LD stage, as shown in a yellow color bar in Figure 6.

**Figure 7:**
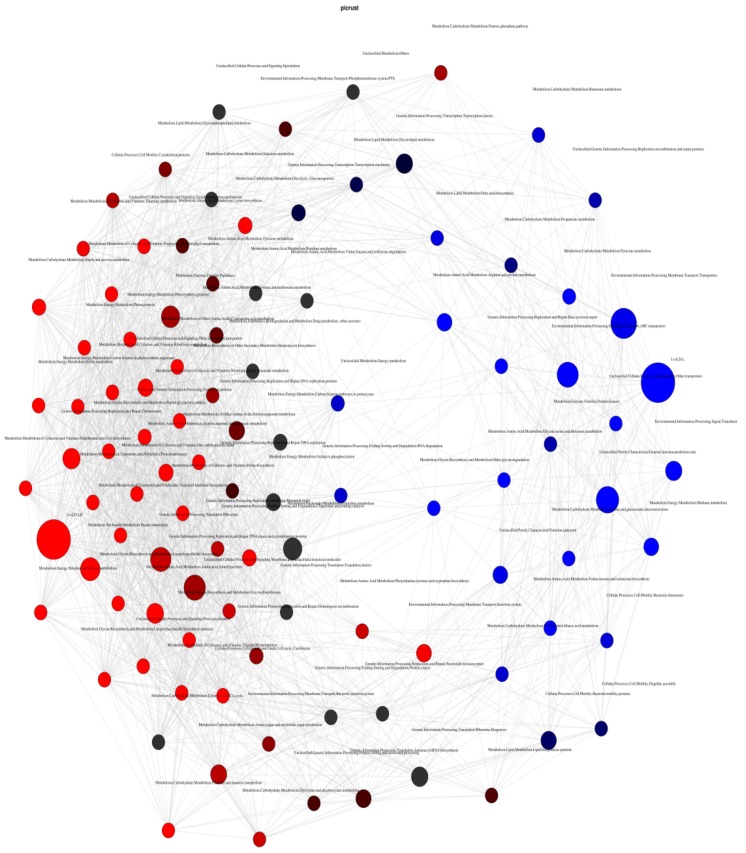
Pearson’s correlation co-occurrence network of microbiota at both stages. Red indicates the L and blue indicates the LD.

Pearson’s correlation network is shown in Figure 7. Based on the top 100 functional pathways, we analyzed the entire predicted metabolic function between the two stages. Nodes have been colored based on their association with selected environmental variables (Pearson’s correlation). L correlations between nodes are presented in red, LD correlations in blue. Pathways involved in the hub node of L are environmental information, membrane transport, and ABC transport, whereas pathways involved in LD are genetic information, DNA replication and repair, and chaperones and folding.

## 4. Discussion

Multiple studies have been published about the microbial composition of the pig gut through high-throughput sequencing technologies as well as culture-based techniques [58,59,60,61]. It was suggested that microbial compositions are directly associated with genetics and aging of swine. It has been reported by multiple researchers that cecum bacterial population particiapates in regulation of harvesting of energy and is associated with body weight [59,62]. To the best of our knowledge, this is the first time there has been an attempt to uncover the microbiota of cecum contents at two different growth stages of Korean native swine. Exploration of bacterial taxonomy composition and their functional pattern at cecum of pig can facilitate an understanding of bacterial impact on host health as well as improving pig production. However, Pajarillo et al. in 2014 compared fecal microbial population between pre-weaning and post-weaning piglets and also established the dominance of *Firmiicutes* and *Bacteroidetes* in both stages [63]. Pigs from weaning stage to 10 weeks (Stage L) are termed as weaners, and from 10 weeks to around 26 weeks (Stage LD) are known as rearing pigs. In this study, 16 samples (8 from each) have been sequenced through 16s rRNA sequencing technologies, offering substantial information regarding their functional behavior. It was observed that at the age of more than 22 weeks, the microbial populations are relatively mature and consistent in cecum content [17]. The population of phyla, such as *Firmicutes* and *Bacteroidetes* are abundant in cecum content in all 16 samples of pig. Other phyla such as *Spirochaetes*, *Protobacteria*, *Verrucomicrobia*, etc. are also present in significant amounts. Whereas *Firmicutes* and *Spirochates* are most abundant in stage L, in contrast *Bacteroidetes* and *Proteobacteria* are most abundant in stage LD. Comparative assesment of OTUs between two growth stages showed that 60 and 43 OTUs are unique in L and LD, respectively. Such relative changes in microbial population may be due to stress or changes in the diet of pigs, as reported previously by Konstantinov et al., 2004, Mackie et al., 1999, and Poroyko et al., 2010 [64,65,66].

Our results are also supported by previous findings, as *Firmicutes* and *Bacteroidetes* were the most abundant phyla in the fecal content of pigs [1,5]. Genus-level comparison showed that *Clostridium sensu stricto 1* and *Treponema 2* are highly abundant in L as compared to LD. These findings also support the research reported by Lamendella et al., 2011 and Le Chatelier et al., 2013 that swine- and human-gut microbiomes are abundant with *Clostridium* and *Treponema* [1,67]. Genus-level univariate analysis also showed that *Prevotella*, *Clostridium*, and *Anaerovibrio* are abundant in swine metagenomes.

Functional annotations through PICRUST of the microbiota are essential to understand their involvement on the host metabolism and disease [68,69]. Purine metabolism (K01524) is differentially enriched in stage L microbiota, whereas ABC-2 type transport system AP-binding protein (K01990) is differentially enriched in stage LD. Kohl et al. in 2018 also reported that purine metabolism is differentially enriched in the cecum of small mammals [70]. Virkel et al. in 2018 [71] reviewed the role of ABC transporters in aging livestock animals through KEGG pathways. ATP-binding cassettes significantly influenced the bioavailability of multiple drugs. Similarly, through PICRUST and STAMP analysis, Yang Hui et al. in 2017 reported that glycine, serine, and threonine metabolism, and valine, leucine, and isoleucine degradation metabolism are significantly abundant [72].

Machine-learning approaches such as Orphelia, MGC, MetaGUN, etc. have been reported for gene prediction and taxonomy classification from metagenome data [73,74,75,76]. However, such approaches are still sparsely used for functional annotations. Here, we explored ANOVA, MixMC, LEfSe, RF, and Pearson’s correlation to establish the functional significance between the two stages of pig cecum metagenome. Yang Hui, et al. in 2016 compared the functional pattern of microbiota of three different gut locations of swine through an ANOVA test [77]. Our ANOVA predictions also indicate that metagenomic functions are linked to the aging of swine. Kouchiwa, Takanori, et al. in 2012 showed that amino acid metabolism is linked to the aging of human, similarly to what we found in the case of the swine metagenome [78]. We used univariate as well as multivariate statistical tools to explore the functional pattern of the microbiome, and found both tools provided similar annotations. MixMC is a well-known multivariate microbial framework that allows annotation of the functional behavior of cecum microbes, and found that amino acid metabolism, cellular processes, and transport systems are abundant in stage L. Similarly in stage LD, glycan biosynthesis, energy metabolism, and nitrogen metabolism are significantly abundant [51].

Ten Zhen et al. in 2017 established the functional capacities with feed efficiency from cecum metagenomic data of swine through LDA effect size. Our results of LEfSe also supported their findings, as some of the metabolic pathways such as energy metabolism are differentially enriched in two different stages of cecum microbiome [79]. Multivariate analysis through PCA and sparse partial least discriminant analysis (sPLS) at both stages have also shown similar metabolic pathways, such as amino acid metabolism, human disease, and genetic information dominating in both stages. Our random-forest classifiers predicted that genetic information, chaperone, and folding pathway, amino acid metabolism, energy metabolism etc. are enriched. Similarly, Maltecca et al. in 2018 carried out random-forest classification technique to decipher the growth prediction and carcass traits by using swine metagenome data [23]. Mach et al. in 2015 and Ramayo-Caldas et al. in 2016 reported that porcine gut microbes are associated with porcine growth, feed efficiency, and enterotype [80,81].

Metabolic functions identified through 16s rRNA analysis are only predictive in nature, as they are probable estimations, based on sequence similarity. Therefore, whole-metagenome analysis along with 16s rRNA analysis is required to know the exact pathways in which the identified microbes are involved. The network analysis may present a tendency of the predicted metabolic function between the two groups. We observed the clear cluster between two growth stages at a functional level through Pearson’s correlation network. Similar analysis on soil microbial communities was reported by Barberan Albert et al., 2012 [82].

## 5. Conclusions

The composition of the cecum microbiome population of swine is strongly related with multiple factors such as developmental stages, immunity, diets, and environmental microbes. We established a critical resource of understanding for the metagenomic implications and bacterial abundance at different times of raising pigs, and emphasize how the microbiome contributes to age-related health. We found *Firmicutes* and *Bacteroidetes* phylum were abundant in both the stages. Interestingly, our findings show that composition of *Bacteroidetes* increases from L to LD, while *Firmicutes* compositions decrease from L to LD. Univariate genus analysis found *Prevotella*, *Clostridium*, and *Anaerovibrio* were significantly abundant. For the first time, we have used multiple machine-learning approaches such as multivariate mixMC, LEfSe, RF, and Pearson’s correlation to explore the functional pattern of microbial populations, and found that amino acid metabolism and energy metabolism pathways were differentially significant at different stages of swine. Pearson’s correlation network showed that genetic information and DNA repair pathways are actively involved while aging in swine. Our report about stage-specific cecum microbial profiling will also be a useful resource in pig health and pig production management systems.

### Nucleotide Sequence Availability

16s rRNA sequences of all 16 samples of this project have been submitted in NCBI SRA (Sequence Read Archive) submission portal and can be accessed through SRA accession PRJNA540190 (https://www.ncbi.nlm.nih.gov/sra/PRJNA540190).

## Figures and Tables

**Table 1:** Feed composition for stage 1 (10 weeks) and stage 2 (26 weeks), Ca: Calcium, P: Phosphorus, DE: Digestible energy, DCP: Digestible crude protein.

Samples	Protein	Crude Fat	Ca	P	Crude Fiber	Crude Ash	Lysine	DE	DCP
Stage 1 (L)	19.0	6.7	0.4	1.5	4.0	8.0	1.40	3.6	16.0
Stage 2 (LD)	14.5	5.5	0.4	1.2	7.0	8.0	0.80	3.5	12.0

**Table 2:** Result summary of Assembly (by FLASH), Q20 (%): The percentage of bases in which the phred score is above 20; Q30 (%): The percentage of bases in which the phred score is above 30.

Sample Name	Sex	Total Bases	Read	GC (%)	Q20 (%)	Q30 (%)
K4.4L	F	86,303,564	189,866	53.08	98.54	95.24
K4.10L	M	100,659,045	223,531	53.1	98.56	95.22
Y5.2L	F	78,466,731	172,363	52.8	98.51	95.14
Y5.3L	M	79,260,896	175,057	53.03	98.55	95.26
Y5.4L	F	74,925,854	165,186	53.18	98.58	95.33
Y5.6L	M	85,490,768	189,176	53.11	98.52	95.06
Y5.9L	M	93,195,851	206,722	53.3	98.55	95.25
Y5.12L	F	92,420,536	204,127	53.21	98.63	95.43
K4.5L.D	M	70,633,514	155,507	53.32	98.62	95.35
K4.8L.D	F	77,651,573	170,983	53.21	98.62	95.47
Y5.1L.D	F	74,716,039	164,985	53.61	98.78	95.75
Y5.5L.D	F	81,054,520	178,286	53.31	98.78	95.8
Y5.7L.D	F	76,811,356	168,951	53.58	98.56	95.29
Y5.8L.D	M	85,996,517	188,902	53.44	98.54	95.29
Y5.10L.D	M	70,647,269	155,842	53.62	98.52	95.27
Y5.11L.D	M	62,782,136	138,382	53.68	98.65	95.46

**Table 3:** Summary of community richness and diversity of all samples from both growth stages.

Sample Name	OTUs	Chao1	Shannon	Simpson	Goods Coverage
K4.5LD	349	399.833	5.999	0.961	0.995
K4.8LD	279	303.117	4.707	0.891	0.997
K4.4L	454	509.122	6.214	0.966	0.997
K4.10L	476	509.552	6.298	0.973	0.998
Y5.1LD	360	405.217	5.941	0.967	0.996
Y5.5LD	381	422.576	6.108	0.973	0.999
Y5.7LD	362	399.631	5.827	0.960	0.996
Y5.8LD	326	355.684	5.355	0.935	0.998
Y5.10LD	373	419.941	6.284	0.975	0.996
Y5.11LD	370	406.875	6.031	0.965	0.995
Y5.2L	418	456.478	5.756	0.958	0.997
Y5.3L	367	410.807	4.938	0.907	0.997
Y5.4L	382	429.275	6.051	0.973	0.997
Y5.6L	397	458.386	5.664	0.954	0.996
Y5.9L	310	351.437	4.998	0.923	0.998
Y5.12L	453	498.122	6.045	0.963	0.997

## Supplementary Materials

The following are available online at https://www.mdpi.com/2073-4425/10/5/382/s1. Figure S1: Rarefaction plots of 16S rRNA amplicons for both stage samples; Figure 2. Univariate analysis through ANOVA; Figure 3. Plots of PCA, CCA, NMDS, RDA showing the multivariate statistical analysis; Figure 4. Differentially abundant selected metabolic activity and their association with stages L and LD through; Supplementary excel sheet 1–4.
