# Supplementary material for: Comparison of Bacterial Populations in the Ceca of Swine at Two Different Stages and Their Functional Annotations
Source: Genes (Basel). 2019 May 20;10(5):382. doi: 10.3390/genes10050382 (PMC6562920; doi:10.3390/genes10050382)
Supplement: Supplementary file 1 [file genes-10-00382-s001.zip › SupplimentaryMaterials/SupplimentaryFigures.docx]

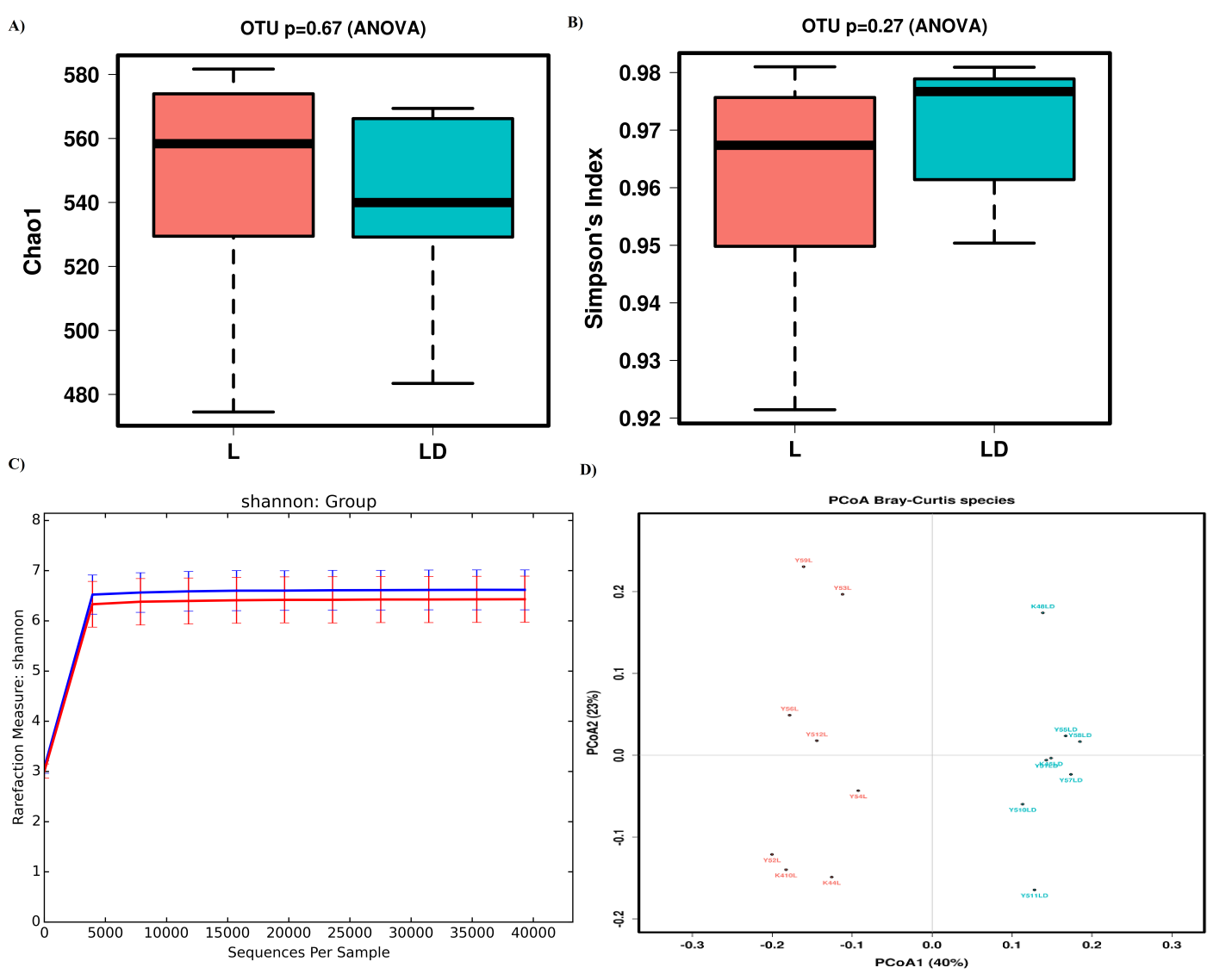


Figure 1. Rarefaction plots of 16S rRNA amplicons for both stage samples a) Chao1 box plot, b) Simpson’s index box plot, c) Shanon index plot, d) PCoA plot showing the differences in cecum microbiota between stage L and LD. L (Red), LD (Blue).


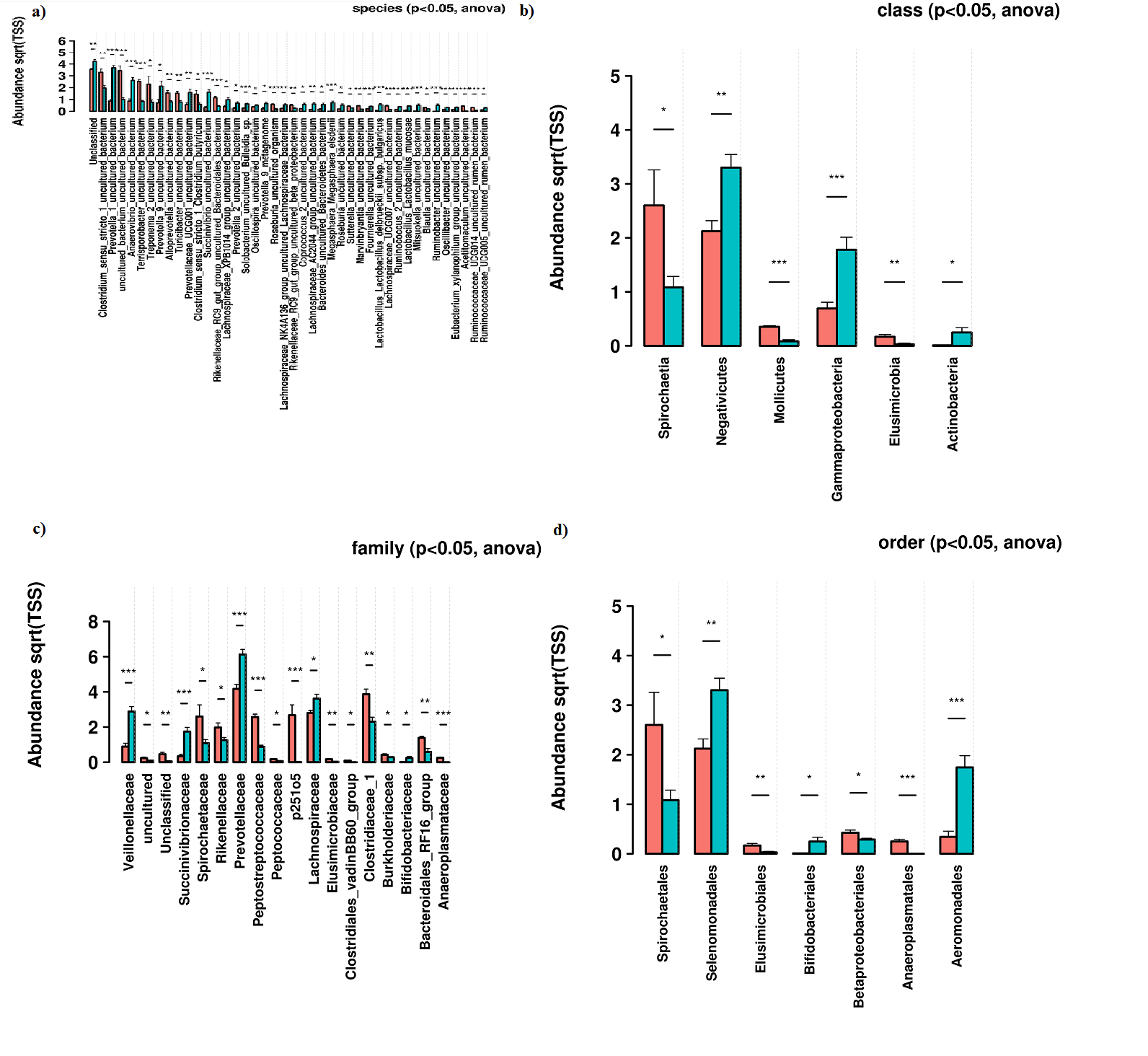


Figure 2. Univariate analysis through ANOVA , Red color indicates the stage L, and blue is stage LD. X-axis representing the samples and Y-axis representing the relative OTU counts. a) Species b) Class, c) Family, d) Order level taxonomic annotation.


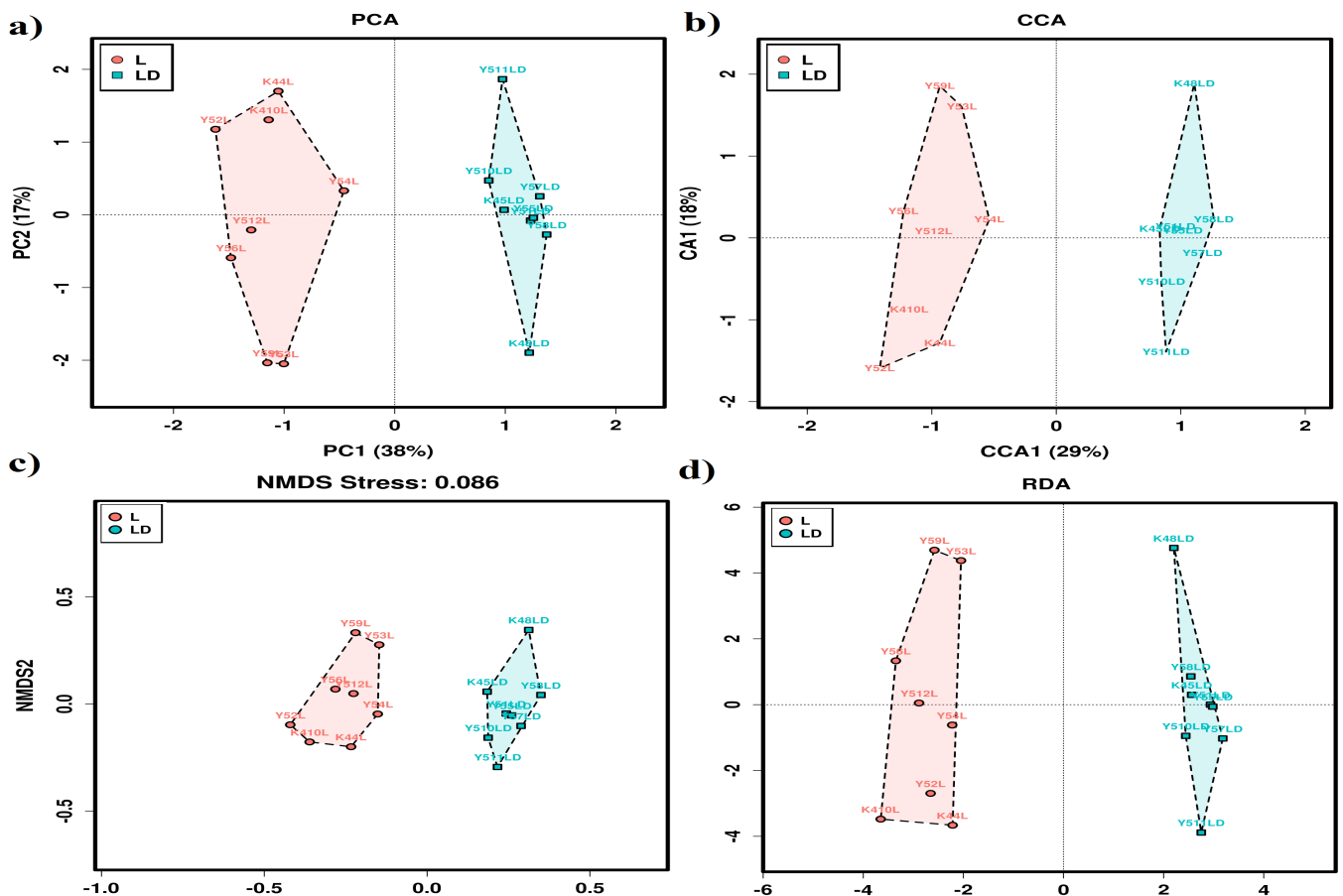


Figure 3. Plots of PCA, CCA, NMDS, RDA showing the multivariate statistical analysis


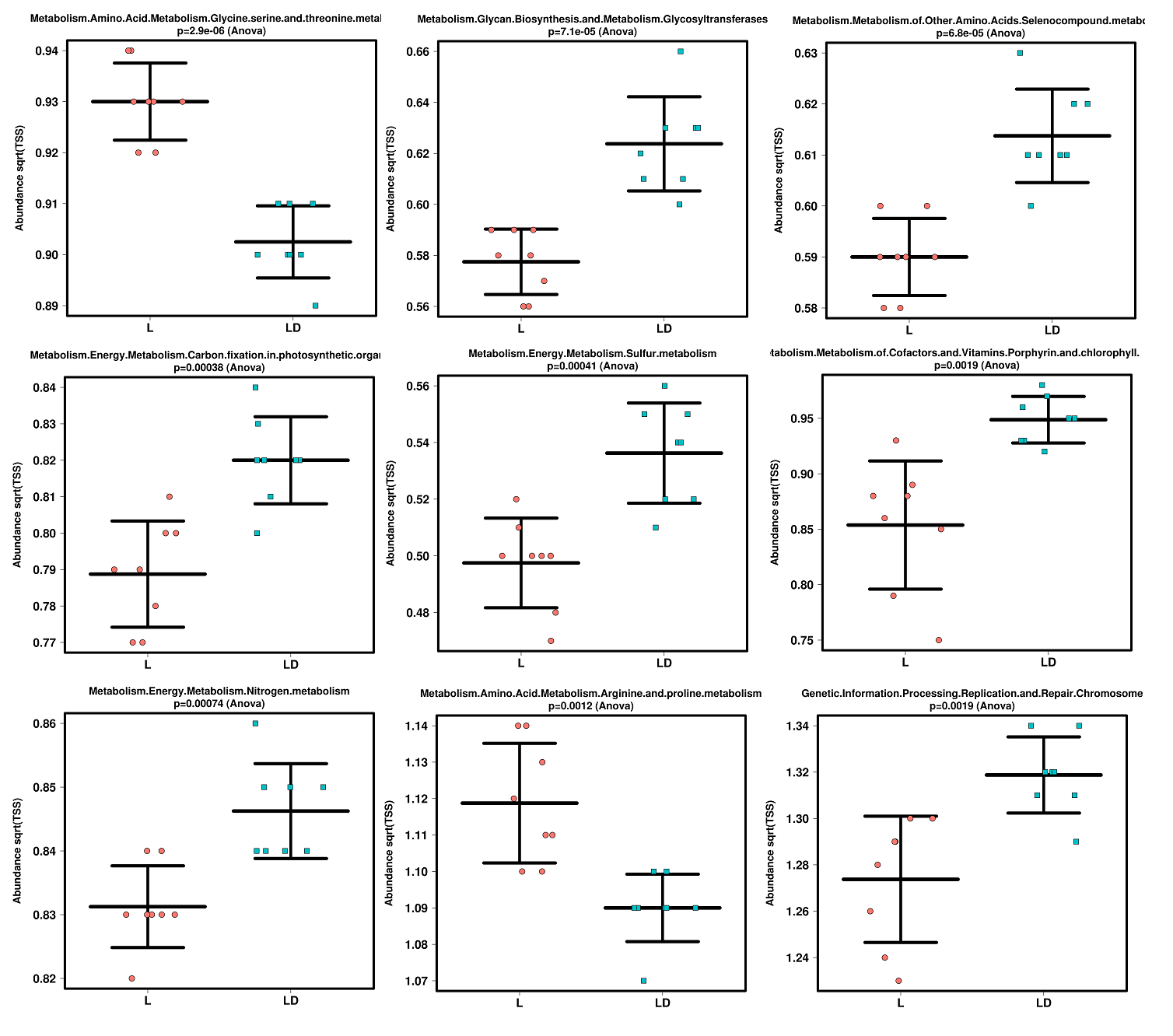


Figure 4. Differentially abundant selected metabolic activity and their association with stages L and LD through


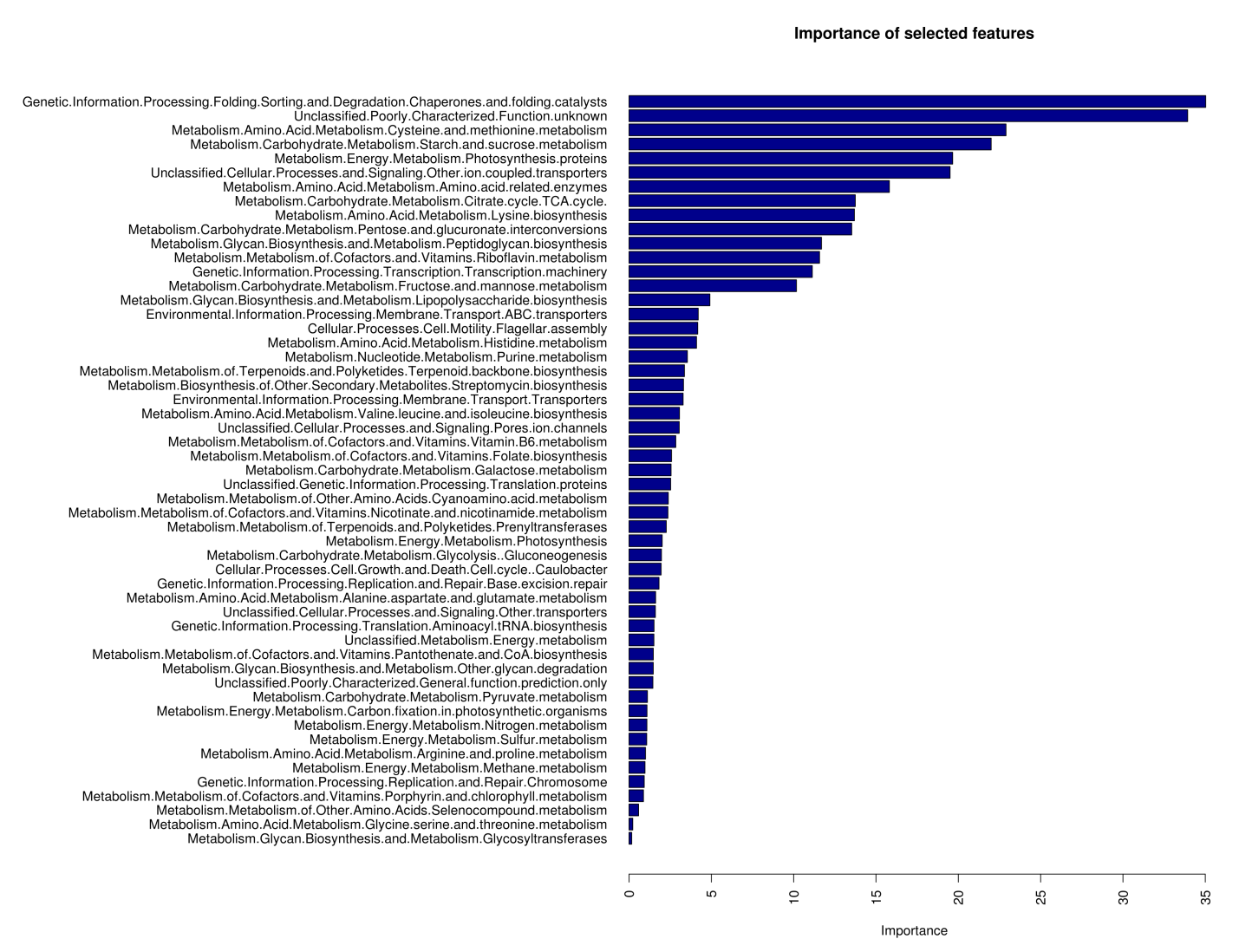


Figure 5. Functional profiling through feature selection by Random Forest method.
